# Book Reviews

**Published:** 1889-06

**Authors:** 


### BOOK REVIEWS.

A Manual of Diseases of the Nervous System. By W. R. Gowers, M.D., F.R.C.P. American Edition, issued under the supervision of the author, and containing all the material of the two-volume English edition, with some additions and revisions. Three hundred and forty-one illustrations. Philadelphia: P. Blakiston, Son & Co.

This work of 1,356 pages is an extension of a work on the Diseases of the Spinal Cord and Nerves which has been for some months before the English part of the profession. The name of Dr. Gowers is too well known to need any introduction. It is not necessary to present the reader with a general plan of the work, as the title gives it sufficiently. The whole subject is handled in a masterly manner, and will not fail to give profit to those who are not abreast with the literature, and satisfaction to those who are. The reviewer is not acquainted with any book in the English language on the diseases of the Nervous System so complete and satisfactory.

It would seem, however, the general literature relating to the chorda tympani has not been as thoroughly examined as we had a right to expect. In proof of this, the paragraph on taste (p. 651) has been compared with the extensive article by Dr. Ed. Schulte, of Berlin, translated by Isidore Furst, of New York, and published in the Archives of Otology, volume XV., p. 62. It is practically certain that the article referred to has escaped the author's notice, or some mention would have been made of it as well as of the works of Sapolini that Dr. Schulte refers to so extensively.

An Elementary Treatise on Human Anatomy. By Joseph Leidy, M.D., LL.D., Professor of Human and Comparative Anatomy and Zoology in the University of Pennsylvania, etc., etc. Second edition, rewritten, with four hundred and ninety-five illustrations. Pp. 950. 1889. Philadelphia: J. B. Lippincott Company. Chicago: W. T. Keener.

After the lapse of twenty-eight years, Professor Leidy has had issued a second edition of his work on human anatomy, revised and rewritten. In this work, as in his teaching of the subject, he has sought to simplify, and thus aid the student of anatomy in his efforts to secure a clear and practical knowledge of the subject instead of simply committing to memory names, some of which are known only by the names of persons. The substitution for such arbitrary names of some that are simple and expressive of the character of the part or of its function is a material aid to the student.

The work of Professor Leidy, either as a teacher or a writer, is always so well and so thoroughly done, that the work now presented needs no higher compliment than the statement that it is characterized by his usual thoroughness. In the dress in which the publishers have issued it, it is a welcome addition to one's library.

Diphtheria: its Nature and Treatment. By C. E. Billington, M.D., and Intubation in Croup, and other Acute and Chronic Forms of Stenosis of the Larynx. By Joseph O'Dwyer, M.D. Octavo, 326 pages. Price, muslin, $2.50. New York: William Wood & Company.

The Author says in his preface "I have, however, endeavored to present a clear and succinct statment of those facts in existing knowledge which are most essential to the formation of an intelligent opinion as to its nature, and of those therapeutical principles and details, the comprehension and application of which will, as I believe, enable the physician to treat it most successfully." The effort has been successful, and the profession is already aware of the good work done by the author in introducing the practice of intubation, with its beneficial results.

Wood's Medical and Surgical Monographs.

The additional numbers for April, May and June have been issued. The April number contains two papers, one "On Diabetes, and its Connection with Heart Disease," by Dr. J. Mayer; and the other on "Blenorrhœa of the Sexual Organs, and its Complications," by Dr. E. Finger.

The May number, two papers also: "On the Preventive Treatment of Calculous Disease, and the Use of Solvent Remedies," by Sir H. Thompson; and "Sprains: Their Consequences and Treatment," by Dr. C. W. M. Moulton.

The June number contains a translation of Dr. A. Schreiber's work on "General Orthopedics, including Surgical Operations," and an index for Volume II.

A Manual of Diseases of the Ear, for the Use of Students and Practitioners of Medicine. By Albert H. Buck, M.D., Clinical Professor of the Diseases of the Ear, in the College of Physicians and Surgeons, New York; Consulting Aural Surgeon, New York Eye and Ear Infirmary. 420 pages. Illustrated. Price, extra muslin, $2.50 New York: William Wood & Company. 1889. Chicago: W. T. Keener.

The present volume is the result of a thorough revision of the author's "Diagnosis and Treatment of Ear Diseases" published in 1880. It is prepared with the thoroughness which characterizes all of the author's work. It is very profusely illustrated, and is a valuable addition to the text-books upon the subject.

A Guide to Therapeutics and Materia Medica. By Robert Farquharson, M.P., M.D., Edin., etc., etc. Late Lecturer on Materia Medica, at St. Mary's Hospital Medical School, etc. Fourth American, from Fourth English Edition. Enlarged so as to include all preparations officinal in the U. S. Pharmacopœia, by Frank Woodbury, A.M., M.D., etc., etc. Philadelphia: Lea Brothers & Co. 1889. Chicago: A. C. McClurg & Co.

This new edition is an improvement on the previous editions, already well known to the medical profession.

The American editor has made in the appendix such additions as will give it additional value to American practitioners and students of medicine.

EXTRA-UTERINE PREGNANCY. A DISCUSSION REPRINTED FROM THE TRANSACTIONS OF THE AMERICAN ASSOCIATION OF OBSTETRICIANS AND GYNECOLOGISTS. Vol. 1, 1888. With an appendix reviewing Mr. Lawson Tait's "Ectopic Gestation and Pelvic Hematocele." Price 75 cents. Pp. 65 and three plates. Philadelphia: William J. Dornan. 1889.

I. *Its Pathology.* By Franklin Townsend, M.D.
II. *Its Diagnosis.* By Joseph Price, M.D.
III. *Its Treatment.* By E. E. Montgomery, M.D.
IV. *Observations—Clinical, Pathological and Surgical.* By W. H. Wathen, M.D.
V. *A Critique of its Management.* By J. M. Baldy, M.D.
VI. *The Technique of the Operation.* By John B. Deaver, M.D.
VII. *Its Management when the Fetus Survives Tubal Rupture and goes on to the Period of Viability.* By L. S. McMurtry, M.D.
VIII. *Its Treatment* (*concluded*). By A. Vander Veer, M.D.

ELEMENTS OF HISTOLOGY. By E. KLEIN, M.D., F.R.S., Lecturer on General Anatomy and Physiology in the Medical School of St. Bartholomew's Hospital, London. Illustrated with one hundred and ninety-four engravings. New and enlarged edition. Pp. xii.—368. 1889. Philadelphia: Lea Brothers & Co. Chicago: A. C. McClurg & Co.

In this, the fourth edition of Klein's histology, issued as one of the *Manuals for Students of Medicine*, the author has brought the work down to the present date, and has sought to have it present the most recent advances made in our knowledge of histology. The illustrative cuts are numerous, and fairly well executed, on fair paper, not well bound.

A MANUAL OF DIETETICS FOR PHYSICIANS, MOTHERS AND NURSES. By W. B. PRITCHARD, M.D. New York: The Dietetic Publishing Company. Pp. 88. 1889.

This manual contains useful information covering various subjects regarding the welfare of human beings of all periods of life, in health as well as in disease.

Whilst most of what is said is, or ought to be, known to the physician, yet it is not known to the laity, and in the convenient and cheap form in which the book is presented to the public, it will no doubt find a ready sale, and serve a good purpose, for none know better than do physicians the lamentable amount of ignorance on the part of people, generally, regarding the laws of health, and how it should be cared for. The perusal of this manual will furnish much useful information that may be advantageously applied.

HANDBOOK OF MATERIA MEDICA, PHARMACY, AND THERAPEUTICS. COMPILED FOR THE USE OF STUDENTS PREPARING FOR EXAMINATION. BY CUTHBERT BOW[illegible]N, M.D., B.A., Editor of "Notes on Prac[illegible]e." 8vo. Pp. vi–366. Philadelphia and London: F. A. Davis, Publisher. 1889.

This handbook was seemingly written expressly for those wh[illegible] do not otherwise enjoy adequate quiz faciliti[illegible]s.

It is an epitome compiled in the form of a question and answer book.

If used by students strictly in accordance with the intention of the author, in connection with and supplementary to the regular textbooks, and not as a substitute therefor, the publication may serve a useful purpose.

THE BACTERIA IN ASIATIC CHOLERA. BY E. KLEIN, M.D., F.R.S. Pp. xv and 179, 12mo. London and New York: MacMillan & Co. 1889. Chicago: W. T. Keener. Price $1.25.

The present volume is a reprint of some articles published in *The Practitioner* in 1886 and 1887. Contributions which have since been made to the knowledge of the subject are added to the book, and it thus represents the present available knowledge upon its subject. The author still does not believe that the comma bacillus of Koch is the cause of Asiatic cholera. His defense is temperate and thorough.

DISEASES AND INJURIES OF THE EAR: THEIR PREVENTION AND CURE. BY CHARLES HENRY BURNETT, A.M., M.D. Pp. 154. 12mo. Price $1.00. Philadelphia: J. B. Lippincott Company. 1889. Chicago: A. C. McClurg and Company.

This little book is one of the "Practical Lessons in Nursing" issued by the publishers. It is intended for use by non-professional readers. The intention to put into the hands of laymen intelligent but untechnical advice on this subject is of course good, and the author's name is a sufficient guarantee.

PHYSIOLOGICAL NOTES IN PRIMARY EDUCATION AND THE STUDY OF LANGUAGE. BY MARY PUTNAM JACOBI, M.D. Pp. 120, 12mo. New York: G. P. Putnam's Sons. 1889.

The chapters in this book are: An Experiment in Primary Education (2); The Flower and the Leaf: The Place for the Study of Language in a Curriculum of Education. Those who are familiar with the style of the author will find their admiration increased by reading this her last contribution.

Diabetes: Its Cause and Permanent Cure. By Emil Schnee, M.D. Translated by R. L. Tafel, A.M., Ph.D. Pp. 2[illegible] Philadelphia: P. Blakiston, Son & Co. 18[illegible] Chicago: W. T. Keener.

This book is written by an enthusiast whose statements will be read with lively interest by those interested in the su[illegible]ject. He claims to have thoroughly reviewed the literature of diabetes, and his unequivocal statement that the disease is curable will attract attention. The author's residence at Carlsbad and Monaco has given him exceptional opportunities for the observation of the disease which he discusses.

Examination of Water for Sanitary and Technical Purposes. By Henry Leffmann, M.D., Ph.D., and William Beam, M.A. Pp. vi., and 106. 12mo. Philadelphia: P. Blakiston, Son and Company. 1889. Chicago: W. T. Keener.

The title indicates the scope of this volume. Microscopical examination of water-sediments is not treated, and bacteriological examinations are not described, since they call for skill not usually possessed by those for whose use the book is intended.

The Diagnosis and Treatment of Extra-Uterine Pregnancy. By John Strahan, M.D., M.Ch., M.A.O. Pp. 106. Philadelphia: P. Blakiston, Son and Company. 1889. Chicago: W. T. Keener.

This is the William F. Jenks Prize Essay of the College of Physicians of Philadelphia. The arrangement of the essay is into two parts. Part I. The Diagnosis, and Part II. The Treatment of Intra-Uterine Pregnancy. The book is furnished with an Index and Bibliography.

Masso-Therapeutics, or Massage as a Mode of Treatment. By William Murrell, M.D., F.R.C.P. Fourth edition. 12mo. Pp. vii and 236. Price $1.50. Philadelphia: P. Blakiston, Son & Co. 1889. Chicago: W. T. Keener.

This is a book which is a good one in spite of its faults. It contains relatively much useless matter, but also gives a valuable explanation of the therapeutic uses and limitations of the method which it describes.

